# Determination of Free Glycidol and Total Free Monochloropropanediol in Fish and Krill Oil with Simple Aqueous Derivatization and High-Performance Liquid Chromatography–Tandem Mass Spectrometry

**DOI:** 10.3390/foods13152340

**Published:** 2024-07-25

**Authors:** Guangxin Yang, Yunyu Tang, Xiaoxia Liu, Longlong Wang, Lixia Qin, Dan Li, Xiaosheng Shen, Cong Kong, Wenlei Zhai, Essy Kouadio Fodjo, Chengqi Fan

**Affiliations:** 1 Key Laboratory of East China Sea Fishery Resources Exploitation, Ministry of Agriculture and Rural Affairs, East China Sea Fisheries Research Institute, Chinese Academy of Fishery Sciences, Shanghai 200090, China; yangpai0717@163.com (G.Y.); tangyunyu@ecsf.ac.cn (Y.T.); yizhangdaodi@163.com (X.L.); longlongwang2022@163.com (L.W.); foodsmc98@126.com (X.S.); fancq@eastfishery.ac.cn (C.F.); 2 School of Chemical and Environmental Engineering, Shanghai Institute of Technology, 100 Haiquan Road, Shanghai 201418, China; lxqin@sit.edu.cn (L.Q.); dany@sit.edu.cn (D.L.); 3 Institute of Quality Standard and Testing Technology, Beijing Academy of Agriculture and Forestry Sciences, No. 9 Middle Road of Shuguanghuayuan, Haidian District, Beijing 100097, China; zhaiwl@iqstt.cn; 4 Laboratory of Constitution and Reaction of Matter (Physical Chemistry), Université Felix Houphouet-Boigny, Abidjan 22 BP 582, Côte d’Ivoire; kouadio.essy@univ-fhb.edu.ci

**Keywords:** monochloropropanediol, glycidol, *p*-(dimethylamino)phenol, krill oil, fish oil, demulsification

## Abstract

This study introduces a novel method for detecting free glycidol and total free monochloropropanediol (MCPD) in fish and krill oil. Before analysis on high-performance liquid chromatography–tandem mass spectrometry (HPLC-MS), *p*-(dimethylamino)phenol was used for derivatization of these compounds, enabling the sensitive determination of these contaminants. The sample preparation procedure includes a simple, efficient pretreatment using NaCl aqueous solution extraction and C18 sorbent cleanup (for demulsification), distinguishing glycidol from MCPD under varied reaction conditions for derivatization (weak acidic and strong alkaline aqueous environments). This approach shows broad linearity from 1 to at least 256 ng·mL^−1^, improved sensitivity compared to standard GC-MS methods, with the limit of detection (LOD) and limit of quantification (LOQ) for MCPD and glycidol in both oil samples verified at 0.5 ng·mL^−1^ and 1 ng·mL^−1^, respectively. Different from previous HPLC-MS methods for direct detection of glycidol esters or MCPD esters, this is the first HPLC-MS method used for the detection of free glycidol and total free MCPD in edible oil. Furthermore, this method can be potentially developed for glycidol or monochloropropane diol esters, which is similar to the current official methods adopted for indirect detection of these contaminants in different food matrices. Application of this detection method to real dietary supplements (fish oil and krill oil) revealed MCPD residues in fish oil (maximum detected: 32.78 ng·mL^−1^) and both MCPD (maximum detected: 2767.3 ng·mL^−1^) and glycidol (maximum detected: 22.2 ng·mL^−1^) in krill oil, emphasizing its effectiveness and accuracy for assessing contamination in these supplements.

## 1. Introduction

Monochloropropanediol, namely 3-chloro-1,2-propanediol (3-MCPD) and 2-chloro-1,3-propanediol (2-MCPD), and glycidol (Scheme S1) are processed contaminants generated during the refining of edible oil at high temperatures in free or esterified forms [1,2,3]. 3-MCPD and 2-MCPD are often found in hydrolyzed vegetable proteins. Glycidol is associated with both the formation and decomposition of MCPD (including 2-MCPD and 3-MCPD). Their ester forms (MCPDEs) and glycidol esters (GE) are the primary contaminants detected in food products, especially in refined oils and fats. Due to their digestive or metabolic conversion, toxicological evaluation and regulatory monitoring are closely tied to understanding their free forms (MCPD and glycidol) [4,5]. Research suggested their toxicity on the kidneys, liver, and reproductive organs [4]. The International Agency for Research on Cancer (IARC) classifies 3-MCPD as a Group 2B (possibly carcinogenic to humans) carcinogen [6,7]. Glycidol is found to be a genotoxic carcinogen and has been classified as a Group 2A (probably carcinogenic to humans) carcinogen. There are limited studies on the toxicity of 2-MCPD, and no health guidance has been published, although it is structurally similar to 3-MCPD. Given their potential health risks, it is important to have reliable methods for detecting these compounds in food.

The detection of 3-MCPDE and GE in various processed foods has rapidly become a hot topic [8,9]. Although direct detection of these compounds on HPLC-MS has been established in previous studies, these methods are challenging in practical application due to the availability of various standard compounds, compromised sensitivity, the difficulty in sample cleanup, which complicated the whole analysis method [10]. Up to now, indirect methods for the determination of 2-MCPDE, 3-MCPDE, and GE have been officially developed by different official organizations [11,12,13,14,15] in various countries. Most of these official methods detect them with an indirect method. They involve the release of MCPD esters into free MCPD, and GE into glycidol, before further pretreatment for determination. Hence, detecting MCPD (including 2-MCPD and 3-MCPD) and glycidol offers the foundation for measuring MCPDE and GE. These methods use phenylboronic acid (PBA) or heptafluorobutyryl imidazole (HFBI) to prepare volatile derivatives for gas chromatography-mass spectrometer (GC-MS) analysis. Strict reaction conditions, stability of these derivatives, and contamination on analysis instruments often cause trouble in their analysis [13,14,16]. Microwave-assisted derivatization of 3-MCPD with acetophenone was developed for HPLC-UV analysis. This derivatization shows good retention ability on the chromatograph, but relative lower sensitivity compared to most commonly used GC-MS analysis method [17]. A stable determination method for detecting 2- and 3-MCPD and glycidol with simple derivative reactions and liquid chromatography–tandem mass spectrometry (HPLC-MS) could be a preferable and better alternative to these traditional GC-MS methods.

Fish or krill oil-based dietary supplements containing polyunsaturated fatty acids (PUFA) have been extensively developed [18,19] in response to aging populations and the increasing consumer demand for nutritional wellness [20,21]. In the past few decades, only few papers have been published on 3-MCPDE and GE in these dietary supplements, mainly salmon or krill oil [22,23,24,25,26]. In this study, an HPLC-MS method for the determination of free MCPD and glycidol was intended to be developed. Similar to official detection methods for MCPDE and GE, this method was expected to be further applied in their determination as a new indirect detection method to detect contamination through hydrolysis to release free MCPD and glycidol before using this method. As a showcase of the practicality of this method, the free MCPD and glycidol in the fish or krill oil were also aimed at being detected using this method. These data can help us understand the correlation between the free and the ester forms.

A derivatization reaction using *p*-(dimethylamino)phenol hydrochloride was first developed in this work. It produces the same derivative after reaction with 2-MCPD, 3-MCPD (both called MCPD), and glycidol. The reaction of *p*-(dimethylamino)phenol hydrochloride with 2-MCPD, 3-MCPD, and glycidol happens simultaneously under alkaline conditions and cannot be distinguished. However, under weak acidic environments, glycidol can react with *p*-(dimethylamino)phenol hydrochloride without the coincident reaction with MCPD (including 2-MCPD and 3-MCPD). This helps to discriminate these contaminant detections by controlling the experimental conditions. Therefore, the determination methods for free glycidol and total free MCPD can reasonably be explored. The new derivatization method shows mild experimental conditions and excellent stability. Furthermore, pretreatment of samples for extracting free MCPD and glycidol from fish oil and krill oil was developed by a direct extract with NaCl aqueous solution and simple cleanup with C18 sorbent, which effectively removes emulsified oil, avoiding more matrix interference and inaccuracy. Previous direct detection of glycidol or MCPD esters through HPLC-MS has been established; however, these methods are incapable of detecting free glycidol or MCPD due to the deficiency of active ionization groups and poor chromatographic performance. This method first uses HPLC-MS for detecting free glycidol and MCPD, with an engineered derivatization reaction to improve the chromatographic performance and sensitivity of the target compounds. The HPLC-MS analysis is environmentally friendly due to the fewer organic solvents involved in the pretreatment and the derivatization in aqueous solutions. Moreover, this method was further validated and applied to detect total free MCPD and glycidol contamination in fish and krill oil, demonstrating its simplicity and accuracy.

## 2. Materials and Methods

### 2.1. Chemicals and Reagents

Standards of 3-MCPD, 3-MCPD-*d*_5_, and 2-MCPD (purity > 98%) were purchased from Dr. Ehrenstorfer GmbH (Augsburg, Germany). Glycidol (purity > 95%) was obtained from LGC Standards (Teddington, UK). Gycidol-*d*_5_ (1000 mg·L^−1^ in ethyl acetone) was supplied by ANPEl Laboratory Technologies Inc. (Shanghai, China). HPLC-grade methanol (MeOH) was bought from J.T. Baker (Radnor, PA, USA). Deionized water (18.2 MΩ) was purified using a Milli-Q water purification system (Billerica, MA, USA). The stock solutions of 3-MCPD, 3-MCPD-*d*_5_, 2-MCPD, and glycidol were prepared in methanol at 1 mg·mL^−1^ Glycidol-*d*_5_ was prepared at 500 ng·mL^−1^ in methanol. Standard solutions remained stable in the darkness for at least 5 months at −20 °C.

*p*-(dimethylamino)phenol hydrochloride was purchased from Titan Technology (Shanghai, China). The derivatization solution was prepared at 100 mg·mL^−1^ by dissolving *p*-(dimethylamino)phenol hydrochloride in deionized water. C18 solid phase extraction cartridges (500 mg/6 mL) were purchased from Bonna-Agela Co., Ltd. (Tianjin, China). Sodium hydroxide, sulfuric acid, sodium dihydrogen phosphate, and disodium hydrogen phosphate of analytical grade were supplied by Shanghai Acmec Biochemical Co., Ltd. (Shanghai, China). Phosphate buffer (0.2 mol·L^−1^) was prepared by mixing 31.5 mL Na_2_HPO_4_ solution (0.2 mol·L^−1^) and 68.5 mL NaH_2_PO_4_ solution (0.2 mol·L^−1^) to obtain a pH of 6.5. Other phosphate buffers of different pHs (5.8, 6.0, 7.0, and 7.5) were prepared by mixing different volume ratios of the Na_2_HPO_4_ solution (0.2 mol·L^−1^) and NaH_2_PO_4_ solution (0.2 mol·L^−1^) under monitoring with a pH meter.

### 2.2. Sample Preparation

**Figure 1:**
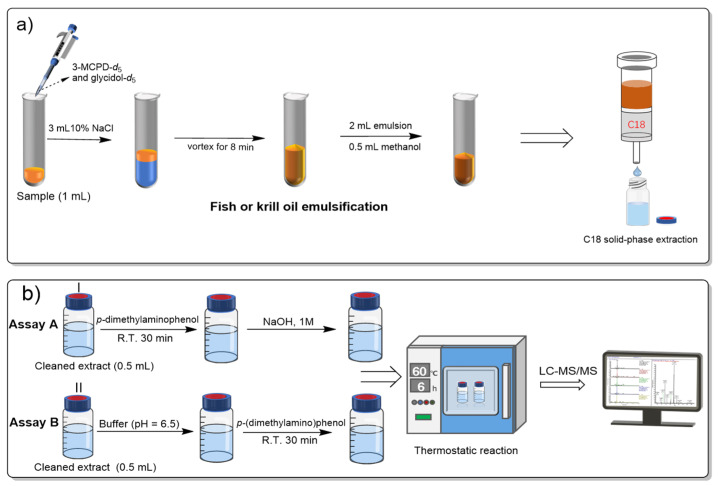
Extraction of MCPD and glycidol from krill oil and fish oil (**a**). Total free MCPD and glycidol derivatization and glycidol derivatization (**b**).

Fish oil and krill oil samples were collected through online purchases or from local supermarkets. These samples were kept at ambient temperature, to be used within their shelf life. Extraction and purification. One milliliter of the fish oil or krill oil was placed into a 10 mL centrifuge tube and spiked with 40 µL of 3-MCPD-*d*_5_ (2 µg·mL^−1^) solution (for total free MCPD and glycidol quantification) and 160 μL of glycidol-*d*_5_ (0.5 µg·mL^−1^) solution (for free glycidol quantification). This sample was then homogenized by vortexing for 30 s. After that, 4 mL of 10% NaCl solution (*w*/*w*) was added to the sample and homogenized by vortexing at 1500 rpm for 8 min. Then, 0.5 mL of methanol was mixed with 2 mL of fish or krill oil emulsion. The solution was passed through the C18 cartridge, and the cleaned extract was collected for the subsequent derivatization reaction (Figure 1a). 

Total free MCPD and glycidol derivatization (assay A). Derivatization began by mixing 0.5 mL of the cleaned extract and 50 µL of the 100 mg·mL^−1^ *p*-(dimethylamino)phenol solution in a 2 mL reaction vial. This mixture was allowed to stand at room temperature for 30 min. Then, 50 µL of 1 mol·L^−1^ NaOH was added to the mixture and homogenized by vortexing at 500 rpm for 1 min. The mixture was kept at 60 °C for 6 h to facilitate the completion of the reaction. Finally, the solution was transferred into 2 mL vials after being filtered with a polytetrafluoroethylene (PTFE) membrane for HPLC-MS analysis (Figure 1b).

Glycidol derivatization (assay B). Approximately 0.5 mL of the cleaned extract was added to a 2 mL vial containing 0.5 mL of phosphate buffer (pH = 6.5, 0.2 mol·L^−1^). Then, 50 μL of *p*-(dimethylamino)phenol hydrochloride solution (100 mg·mL^−1^ in deionized water) was added, thoroughly shaken, and left for 30 min at room temperature before the derivatization reaction for 6 h in an oven at 60 °C. Afterwards, the derivatized solution was filtered with a PTFE membrane for subsequent HPLC-MS analysis. The results of assay B were determined as the amount of glycidol (Figure 1b).

Calibration and quantification for assay A and assay B. Standard solutions of 3-MCPD and glycidol were prepared in a 10% NaCl solution containing 25% MeOH, respectively. Their concentrations were obtained at 1, 2, 4, 8, 16, 32, 64, 128, and 256 ng·mL^−1^. These solutions were all added with internal standards of 3-MCPD-*d*_5_ and glycidol-*d*_5_ at 20 ng·mL^−1^ for quantification. They were derivatized following assay A and assay B, respectively, and were further analyzed through HPLC-MS. The analyte identification was carried out by comparing the retention time (<0.1 min) and the abundance ratios of fragments (212.1/137.1 and 212.1/136.1) between the standards and the samples, which were acquired through parallel reaction monitoring (PRM) mode on the instrument. The chromatogram peak area ratios were obtained for the fragment of the analyte derivative (212.1/137.1) and the internal standard derivative (217.1/137.1). These peak ratios were plotted versus the concentration for calibration curve preparation. The calibration curves for assay A and assay B were used to quantify the analytes. The total amount of 2-MCPD, 3-MCPD, and glycidol was calibrated with the curve of 3-MCPD derivatives, according to assay A. The glycidol content was calculated through the calibration of glycidol derivatives, according to assay B. Then, the total free 3-MCPD and 2-MCPD (MCPD) was calculated by subtracting 110/74*Cglycidol from the total amount result from assay A (110/74, conversion factor of glycidol to equivalent MCPD). 

### 2.3. HPLC-MS Analysis 

HPLC-MS analyses were performed using an HPLC system (DionexUltimate 3000, ThermoFisher Scientific, Waltham, MA, USA) integrated with a quadrupole-orbitrap mass spectrometer (Q-Exactive, ThermoFisher Scientific, USA) using an electrospray ionization source. The derivatized analytes were separated on a C18 column (EclipsePlus C18 RRHD, 3 mm × 150 mm, 1.8 μm) at a flow rate of 0.5 mL·min^−1^ with an injection volume of 4 μL. The column was kept at 35 °C during analysis. The mobile phase A, water (containing 2 mL·L^−**1**^ of formic acid and 5 mmol·L^−1^ ammonium acetate), and phase B, methanol (containing 2 mL·L^−1^ of formic acid), were adopted for HPLC separation. The following gradient elution was applied for chromatographic separation of analytes: 0–1.50 min, 1% B; 1.50–6.00 min, 1–20% B; 6.00–6.15 min, 20–100% B; 6.15–8.50 min, 100% B; 8.50–8.65 min, 100–1% B; and 8.65–10.00 min, 1% B.

The derivatized analytes were ionized in positive mode and analyzed at the following settings: Spray voltage = 3.2 kV; capillary temperature = 350 °C; gas (N_2_) temperature = 400 °C; aux gas flow = 15 arb (arbitrary units); sheath gas flow = 50 arb. Measurements were carried out in PRM mode. Table 1 shows the optimized MS/MS parameters. Data acquisition and analysis were performed with the standard instrument software, Xcalibur Version 3.0 (ThermoFisher Scientific, USA). 

### 2.4. Method Validation and Statistical Analysis

The method was validated following the guide in SANTE 11312/2021 [27], including the limit of detection (LOD), the limit of quantification, and the linearity, accuracy, and stability of the spiking experiment. The LOD and LOQ were determined based on the peak height at approximately 3 and 10 times the baseline noise height, i.e., S/N > 3 and S/N > 10, respectively. For the 3-MCPD spiking experiment, these spiking tests were performed at three levels, including the addition of 5, 20, and 40 ng·mL^−1^ 3-MCPD to fish oil, and another spiking of 200 ng·mL^−1^ 3-MCPD was added to krill oil. These samples were extracted and measured according to assay A. For glycidol spiking experiment, 3 levels of spiking concentration, including 2, 5, and 20 ng·mL^−1^, were spiked in krill and fish oils, respectively. Each of the above-mentioned spiking levels in krill or fish oil was repeated three times. Statistical analysis was carried out by calculating the average values of repeated samples and determinations, in parallel with their relative standard deviations.

## 3. Results and Discussion

### 3.1. Derivatization and HPLC-MS Analysis

The structural properties of MCPD and glycidol suggest less sensitivity to electrospray ionization or electron ionization in mass spectroscopy analysis. In order to facilitate detection via mass spectrometry, it is imperative to establish a link between these molecules and a functional group. Traditionally, these compounds were subjected to derivatization using PBA, HFBI, and other relevant agents to improve their detection sensitivity on a GC-MS apparatus. However, these compounds exhibit a limited response in HPLC-MS analysis. Therefore, additional functional groups were investigated to enhance the sensitivity of electrospray ionization for subsequent MS analysis. This work extensively examined the use of *p*-dimethylaminophenol hydrochloride as a novel derivatizer. Its reactivity with MCPD and glycidol was investigated, as its dimethylamine group is active for electrospray ionization. 

**Scheme 1:**
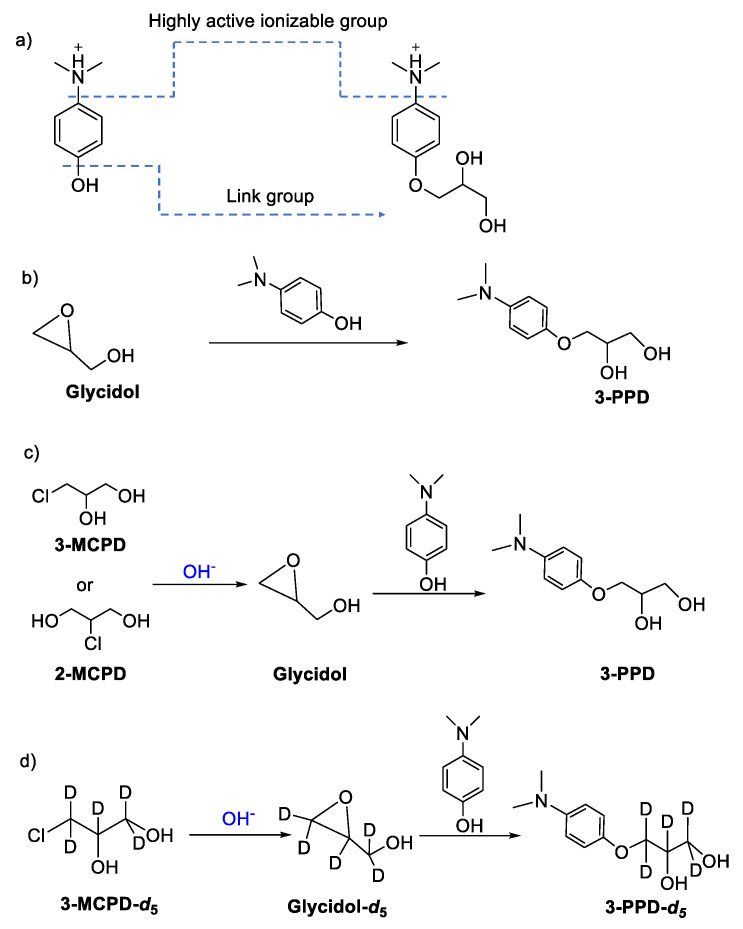
Diagram of (**a**) the highly active ionizable group and link group of derivatives and its product, and synthetic route of derivatization reactions for (**b**) glycidol, (**c**) 3/2-MCPD, (**d**) 3-MCPD-*d*_5_, and glycidol-*d*_5_.

As shown in Scheme 1a, the *p*-(dimethylamino)phenol endows the target analytes with high mass spectrometric activity [28], which usually requires an easy ionizable group and a reaction-active link group. The dimethylamino group is very active for electrospray ionization. The hydroxyl group has a relatively high reactivity and can easily react with epoxy groups on glycidol through nucleophilic attack to produce 3-PPD (Scheme 1b). On the other hand, the MCPD can easily be converted to glycidol under strong alkaline conditions (Scheme 1c). Theoretically, they can also form 3-PPD when reacting with *p*-(dimethylamino)phenol. This final derivative, 3-PPD, has been confirmed through the mass spectrum, 1H NMR, and 13C NMR measurements (Figures S1–S4). 

Furthermore, the secondary fragments of 3-PPD and their isotopic derivatives (3-PPD-*d*_5_) have been acquired and optimized for subsequent HPLC-MS detection. A PRM mode has been implemented to realize a more accurate and short-term analysis, where the fragment ions improved the sensitivity and accuracy. In addition, the complete separation on the column was unnecessary as the PRM mode acquires secondary fragments and enhances the selectivity to discriminate the analyte signal from interfering matrices. The molecular ion peak of 3-PPD in positive ion mode was obtained at *m*/*z* 212.12770, and its fragmentation was obtained at *m*/*z* 137.08331 and *m*/*z* 136.07550. The fragmentation pattern of 3-PPD-*d*_5_ was identical to the above results. Scheme 1d shows the reactions that occur during the synthesis of glycidol-*d*_5_. 

### 3.2. Pretreatment Process Optimization

To extract these analytes at high efficiencies, a pretreatment approach was explored by a simple aqueous extract and subsequent cleanup using a C18 solid-phase extract cartridge (C18 SPE). The approach employed in our study was integrated with the above derivatization reactions, which exhibits enhanced sensitivity for MS measurement. This procedure confers numerous benefits, such as user friendliness, expeditiousness, enhanced accuracy, and sustained stability over extended periods.

The extract process of MCPD and glycidol in fish oil and krill oil dietary supplements was designed with modification, referring to GB 5009.191-2016 [29], with the aim of simplifying the procedure and reducing operation time. Initially, 20% of NaCl solution was tested for its efficiency in extracting MCPD. It was found that these samples would obviously be emulsified and would not form a distinct water–oil interface after 30 min of silence, especially for krill oil. For this reason, the demulsification process was further examined to obtain a clear extract for subsequent derivatization (Figure S5, Table S1). A new method involving passing the emulsified solution through a C18 SPE as this sorbent was effectively tested, which can adsorb lipid components and may further break oil-water emulsions. A clear extract solvent was obtained (Figure S6). The column efficiency of the C18 SPE was tested to be 98.7% for 3-MCPD and 92.4% for glycidol, indicating excellent performance in the cleanup of the emulsification. Detailed information on the process of selecting the approach of demulsification, the optimization of NaCl concentration, and vortex time during extraction is presented in Supplementary Materials (Figures S7–S10). Optimized NaCl concentration (10%) and vortex time (8 min) were finally obtained. As in previous reports concerning the ester bond form rather than free MCPD or glycidol determination in oil samples, the extraction method in our method is quite different from the previous approach of extracting ester bond MCPD and glycidol [10].

### 3.3. Optimization of Derivative Parameters 

The interferences in the sample extract may influence the derivatization reaction, making the reaction rate different from that of a pure solvent. Therefore, after the sample extraction and cleanup method optimization, the derivatization reaction was optimized to obtain a high yield. 

#### 3.3.1. Derivatization Optimization for the Total Amount of 3-MCPD, 2-MCPD and Glycidol

Four parameters, including the concentration of *p*-(dimethylamino)phenol and NaOH, derivatization time, and reaction temperature, were examined for their influence on the yielding rate of 3-PPD (see Supplementary Materials, Figure S11). The optimized parameters for assay A in a practical matrix have been examined at 100 mg·mL^−1^ of *p*-(dimethylamino)phenol, 1 mol·L^−1^ of NaOH, 60 °C, and 6 h, which achieve a high yield reaction of 3-PPD.

Assay A was developed for the determination of the total amount of 3-MCPD, 2-MCPD, and glycidol. Therefore, 2-MCPD and glycidol should also be evaluated with the above-optimized parameters (see Supplementary Materials). Results showed less than 4% deviation in response signals between 3 samples with single analyte spiking, and the sample with all 3 analytes added shows 5% deviation from the sum of the signals with single analyte spiking. It indicated good signal consistency between these analytes, and the derivatization response signals can be used for quantification of each or the sum of them.

#### 3.3.2. Optimization of Glycidol Derivatization

**Figure 2:**
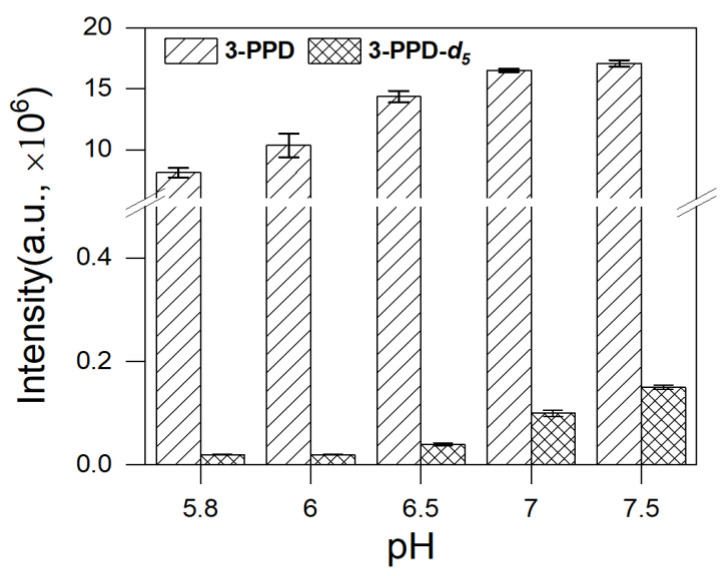
Derivatization of glycidol (100 ng·mL^−1^) and 3-MCPD-*d*_5_ (100 ng·mL^−1^) under different pH tuned by phosphate buffer.

Assay B was developed to determine glycidol. It also needs derivatization, and 3-MCPD should not interfere with quantification. To prevent the conversion of 3-MCPD and glycidol, the pH of the reaction solution should be kept in acidic conditions [28]. We have examined the pH from 5.8~7.5, and the interconversion between these two compounds was confirmed through the response profiles of 3-PPD-*d*_5_ and 3-PPD (Figure 2). It was found that a pH below 6.5 could completely inhibit the reaction of 3-MCPD with *p*-(dimethylamino)phenol, while the addition of glycidol can still result in high response signals for 3-PPD. A lower pH value was found to reduce the reaction yield rate. Therefore, pH 6.5 is kept in subsequent optimization experiments for derivatization of glycidol. Afterward, glycidol was added to the blank oil sample extract and derivatized according to assay B. Three parameters were optimized, including *p*-(dimethylamino)phenol concentration, heating temperature, and time (see Supplementary Materials, Figure S12). The selected derivatization parameters for glycidol were also 100 mg·mL^−1^ *p*-(dimethylamino)phenol at 60 °C in an oven for 6 h without the addition of NaOH.

Both derivatization reactions for assay A and assay B were carried out in mild aqueous conditions. It significantly differs from previously used derivatization agents such as PBA and HPFI, which need organic solvent, or even strict water-free conditions [30]. The derivatization time can be relatively long compared to other derivatization reactions. However, this time is selected to guarantee a high yield reaction, which would not be necessary if isotopic internal standards were applied, and the high detection sensitivity can be realized even within a 0.5 h reaction time [31].

### 3.4. Matrix Effect

To evaluate the cleanup effect, the matrix effect of the extract was tested by spiking 3-MCPD or glycidol in a blank matrix, derivatizing the analytes according to assay A and assay B. These spiked samples were measured on HPLC-MS and compared with the signal in a pure methanol–water (25%) solvent, respectively. The matrix effect was calculated by the ratio of the response values of the derivatized reagent in the blank matrix and solvent added with the same concentration of analytes. In krill oil extract, with assay A, matrix effects of 83% for 3-MCPD, and 96% for glycidol were observed. They are 91% for 3-MCPD and 97% for glycidol in fish oil extract. After derivatization according to assay B, the matrix effect for glycidol was found to be 96% and 97% in krill oil and fish oil extract, respectively. The above results demonstrated a good cleanup effect of the extract using our developed method. However, to further maintain the result stability and offset the deviation during sample preparation, derivatization, and instrument analysis, the internal standards, 3-MCPD-*d*_5_ and glycidol-*d*_5_, were applied in the whole method.

### 3.5. Validation of the Method

#### 3.5.1. Limit of Detection, Limit of Quantitation, and Linear Range

**Figure 3:**
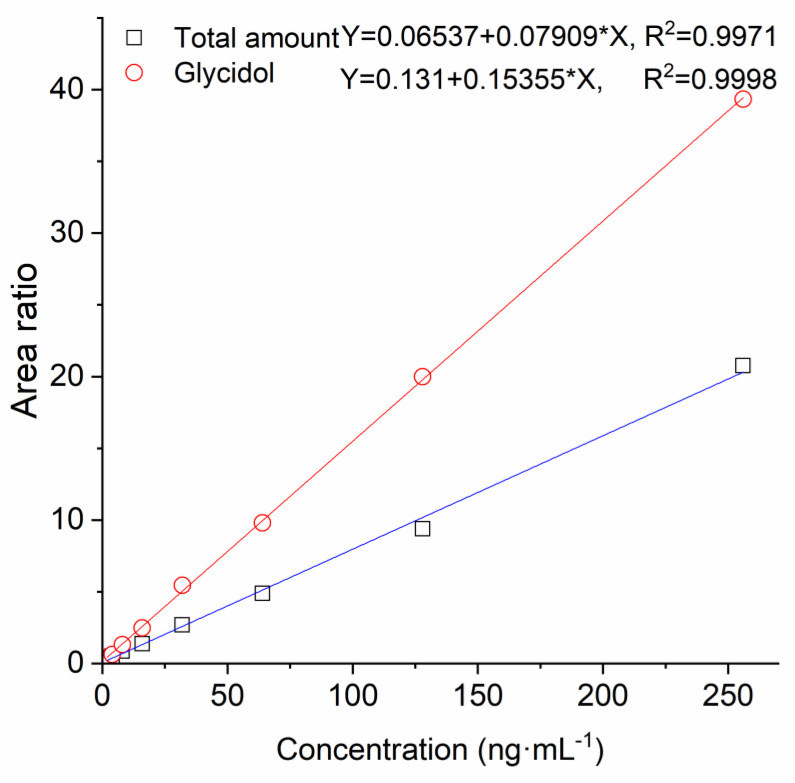
Calibration curves of MCPD and glycidol with 10% NaCl containing 20% MeOH for assay A and assay B.

After the optimization of the derivatization reaction, extraction, and cleanup process, the method’s performance was further evaluated, including LOD, LOQ, and their liner range. Results confirmed that, with assay A, the LODs for the total amounts of 3-MCPD, 2-MCPD, and glycidol in fish oil and krill oil were all at 0.5 ng·mL^−1^, and their LOQ were all at 1 ng·mL^−1^. Similarly, the LOD of 0.5 ng·mL^−1^ and LOQ of 1 ng·mL^−1^ for glycidol were also validated with assay B. Our study significantly improved the sensitivity for the detection of 3-MCPD compared with the Chinese national standard method (GB 5009.191) and the method published by EFSA (the 2017 EFSA method) [31,32], respectively. Furthermore, this study assessed the calibration range for assays A and B, respectively. A linear range of 1~256 ng·mL^−1^ was obtained for both calibration curves in krill or fish oil, with the coefficient of determination > 0.995, indicating excellent linearity of 3-PPD formed through derivatization of MCPD and glycidol using assay A or assay B (Figure 3). 

#### 3.5.2. Recovery and Precision

In this study, method performance was also evaluated based on its recovery and precision. Blank fish oil and krill oil were selected for spiking experiments with 3-MCPD and glycidol. Results showed that the mean recoveries of 3-MCPD in the two matrices varied between 96.3% and 117.4%, with relative standard deviations (RSDs) ranging from 1.45% to 3.93% (Table S2). To check the inter-batch stability, these three spiked levels in fish oil and one in krill oil were repeatedly tested for three consecutive days. Results showed that the mean inter-batch recoveries of 3-MCPD in the two matrices varied between 98.7% and 111%, with RSDs ranging from 1.77% to 13.5% (Table S3). In this study, the 2-MCPD and 3-MCPD were determined in parallel, and the result was calculated as their total amount. Their derivatization efficiency and extraction efficiency were comparable in real sample tests. Therefore, further spiking experiments were carried out to see the synchronization of 2-MCPD and 3-MCPD in the whole method. Our result demonstrated that 2-MCPD and 3-MCPD recoveries in spiking experiments at the same concentration level remained highly consistent (Table S4). This result indicated that 2-MCPD and 3-MCPD were highly synchronized in the experiment, including pretreatment and derivatization. Furthermore, the average recoveries of glycidol in the two matrices varied between 91.6% and 118.6%, with RSDs ranging from 2.0% to 6.2% (Table S5). These spiking experiments were also repeated on three different days, and the inter-batch average recoveries of glycidol were validated to vary between 98.97% and 113.19%, with RSDs ranging from 1.25% to 9.31% (Table S6). The above spiking results demonstrated that current methods have excellent accuracy and precision in both fish oil and krill oil.

#### 3.5.3. The Effect of NaCl Solution on Glycidol

The interaction of chloride ions with epoxy-propanol or cyclic acyl oxygen ions in the processing of glycerol and acylglycerol as precursors is critical for generating MCPD (including 2-MCPD and 3-MCPD). This study examined whether the presence of NaCl in the extract may react with glycidol to form 3-MCPD during the measurement, interfering in 3-MCPD quantification. In the liquid–liquid extraction process with NaCl aqueous solution, whether the extract and glycidol would react with chloride ions during pretreatment to convert glycidol to MCPD was investigated. Three sets of comparison experiments were done under the same spiked glycidol concentration as shown in Table S7. Results showed no significant difference in the signal ratios (3-PPD/3-PPD-*d*_5_) between the above three solutions (Figure S13). Therefore, it is confirmed that the presence of NaCl would not affect the glycidol quantification.

#### 3.5.4. The Effect of MCPD on Glycidol

Based on the formation mechanism of 3-MCPD and its esters, 2-MCPD is a contaminant that appears along with 3-MCPD and glycidol during food processing. Therefore, the possible interference from 2-MCPD and 3-MCPD conversion to glycidol should also be examined in assay B to check whether the phosphate buffer used inhibits the conversion between 2-MCPD, 3-MCPD, and glycidol, resulting in a false-positive or over-100% recovery determination.

**Figure 4:**
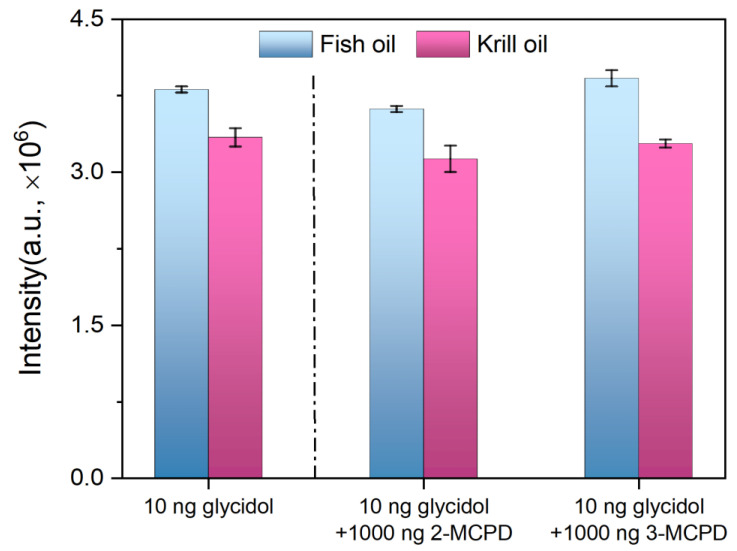
Effect of MCPD presence on the glycidol determination in fish oil and krill oil.

In this work, the blank fish oil and krill oil sample matrices were prepared according to the method of assay B. At the spiking level of 10 ng·mL^−1^ of glycidol in krill and fish oil, 1000 ng·mL^−1^ of 3-MCPD in these samples were added in order to see the accuracy of glycidol quantification in the presence of 3-MCPD, which was measured following assay B. This means equal amounts of glycidol were added to all three blank samples, and 2-MCPD or 3-MCPD were added at a concentration 100 times higher than glycidol in two of the three samples, respectively. All three samples were derivatized according to the method of assay B. It showed that the signal response values of all three samples were similar to each other (Figure 4). The spiking level in both matrices in the presence of 100 times 3-MCPD displayed a glycidol quantification result that was no more than 3.4% higher than that with only glycidol addition. It indicated that the phosphate buffer (pH = 6.5) could well inhibit the conversion of MCPD to glycidol and further prevent the derivatization reaction of MCPD, therefore guaranteeing the selectivity of assay B for glycidol determination.

### 3.6. Method Comparison

As validated in the above sections, the method in this work can achieve a sensitive detection of total free MCPD and glycidol, which can perform better or be comparable to the published reports. There is scarce work on the detection of free glycidol in different food matrices, and the detection of free MCPD in fish or krill oil is also less reported or focused. Therefore, recently published work on the derivatization determination of 3-MCPD and released glycidol from its esters in different food matrices is compared in Table 2. These methods are based on detection with the currently popular derivatization reaction, and GC-MS or HPLC-UV analysis. The developed method exhibited a higher detection limit, with a facile derivatization reaction in the aqueous phase, and realized the extraction through liquid–liquid extraction and demulsification with C18 sorbent. However, it should be acknowledged that the developed method could not discriminate between 2-MPCD and 3-MCPD according to their derivatization mechanisms. Even though the detection of glycidol is reliable, it can be further employed to detect its ester form, referencing the official analysis method. The total free MCPD can represent, to some extent, the contamination level of chloropropandiol in food matrices.

### 3.7. Real Samples Analysis

**Figure 5:**
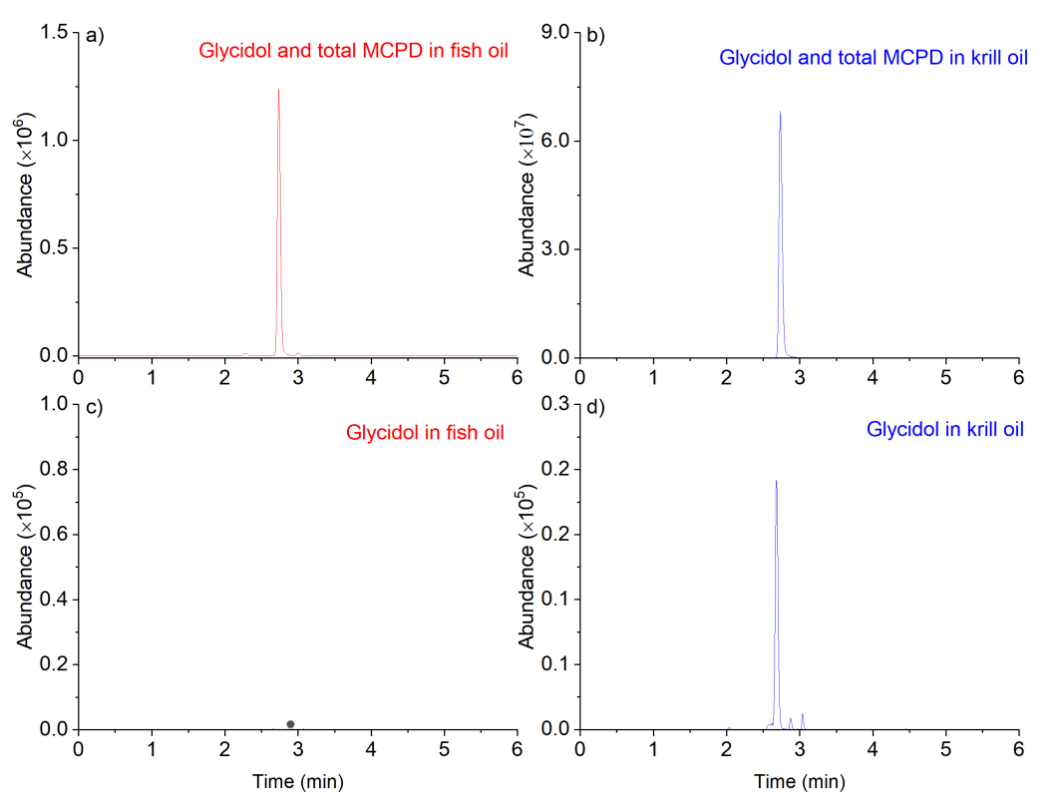
Representative extracted ion chromatography of MCPD and glycidol contaminated fish oil (**a**,**c**) and krill oil (**b**,**d**) samples.

As an alternative method for the determination of MCPD (including 2-MCPD and 3-MCPD) and glycidol in krill oil and fish oil, our developed method has been preliminarily applied to real samples to investigate the contamination of free MPCD and glycidol. Ten fish oil and eight krill oil samples of different brands were purchased from local and online supermarkets. All products experienced extract and cleanup steps before derivatization, according to assay A and assay B, respectively. Representative extracted ion chromatography of blank and positive fish and krill oil samples is displayed in Figure 5. Results showed that the highest level of MCPD contamination in fish oil was 32.78 ng·mL^−1^, and 3-MCPD was not detected in three fish oil and one krill oil samples (Table S8). In contrast, the contamination level of MCPD in krill oils was found to be extensively much higher than that in fish oil, with the highest level of 2767.3 ng·mL^−1^ (Table S8). This may imply that some critical components or different refinery processes exist between the fish and krill oil, playing a significant role in the contamination of MCPD. Another difference could be the contamination of krill and fish oil. In terms of glycidol contamination, it happens rarely in the fish oil samples, as no glycidol was detected positive in these fish oil samples, while no more than 22.2 ng·mL^−1^ of glycidol contamination level was found in these krill oil samples (Table S8). The previous report displayed the occurrence of ester bond MCPD and glycidol in krill oil and fish oil samples. However, different from the contamination profile-free MCPD and glycidol, these ester bond contaminants were found to be more abundant in fish oil samples [22]. These contamination differences are worth studying to find the factors that enhance or inhibit the presence of MCPD and glycidol in these samples. As fish oil and krill oil are some of the favorite choices of dietary supplements for kids, adolescents, and seniors, sufficient attention needs to be paid to these hazardous contaminants for human health.

## 4. Conclusions

In this work, a novel HPLC-MS method for the determination of total free MCPD and free glycidol in fish oil or krill oil was first proposed based on a highly sensitive derivative from these compounds. The extraction of target molecules was carried out through a simple NaCl aqueous solution, and cleanup was performed by passing the extract solution through a C18 sorbent SPE to remove the emulsion in the extract. Afterward, the cleaned extract was derivatized with *p*-(dimethylamino)phenol to form a new derivative for these targets, which is highly sensitive and easy to analyze on an HPLC-MS instrument. The involved reaction derived the same compound for MCPD and glycidol. The free glycidol can be determined directly using the controlled derivatization reaction in the method. The optimized pretreatment and derivatization methods are simpler and more sensitive. Previous HPLC-MS methods directly detected ester bond MCPD and glycidol without derivatization. However, these methods cannot be applied to detecting free MCPD and glycidol due to the deficiency of active ionization groups and poor chromatographic performance. Compared with traditional GC-MS methods, the derivatization process is not susceptible to water content. The resulting derivatives are stable and can be analyzed directly on the HPLC-MS instrument. This determination method is expected to be extended to other matrices, such as plant oil, fish meal, frying and baking, etc. However, the 2-MCPD and 3-MCPD could not be distinguished using our method, which limited the application in some situations. Future work would be focused on how to quantify 2-MCPD and 3-MCPD, individually.

Moreover, this method can be further used in the determination of ester bond MCPD and glycidol after thoroughly examining the compatibility of different hydrolysis parameters. Additionally, our findings illustrate the prevalent contamination of free MCPD and glycidol in krill oil, which exhibits notable differences in contamination levels compared to fish oil. This difference justifies further investigation to ascertain the underlying causes and identify the crucial factors influencing the formation of MCPD and glycidols during the refining process of these oils. Consequently, this knowledge can be effectively utilized to implement measures to control the occurrence of these contaminants.

## Figures and Tables

**Table 1:** MS/MS parameters of PPD (3-MCPD, 2-MCPD, glycidol derivatives) and PPD-*d*_5_ (their isotopic internal standards derivatives).

Compounds	Precursor Ion (*m*/*z*)	Product Ions (*m*/*z*)	CE (eV)
PPD (MCPD/glycidol derivative)	212.1	137.1 ^a^	20
		136.1	40
PPD-*d*_5_ (MCPD-*d*_5_/glycidol-*d*_5_ derivative)	217.1	137.1	20

^a^ Quantification ion.

**Table 2:** Comparison of the recently published methods on the detection of chloropropandiol and glycidol in different food matrices.

Target Compound	Matrices	Derivatrization Agent	Solvent	LOD/LOQ	Instruments	References
2/3-MCPD and glycidol esters (calculated as 2-MCPD, 3-MCPD, and glycidol)	Vegetable oil	HFBI (glycidol was converted to 3-MBPDE first)	n-hexane	5, 2, 10 (μg·kg^−1^, expressed as 2-MCPD, 3-MCPD and glycidol, individually)	GC-MS/MS	[14]
3-MCPD	Rats	HFBA	Ethyl acetate	0.8 μg·kg^−1^	GC-MS	[33]
3-MCPD	HVP	PBA	Aqueous (NaCl solution)	3.87 μg·kg^−1^	GC-MS	[34]
3-MCPD	Soy sauce	Acetophenone	Toluene	80 ng·mL^−1^	HPLC-UV	[17]
3-MCPD	paperboard food packaging	BSTFA and TMCS, 99:1	Acetone	10 μg·kg^−1^	GC-MS	[35]
3-MCPD	Edible oil	TMSI	Ethyl acetate	0.6 μg·kg^−1^	GC-MS/MS	[36]
2/3-MCPD in total, and glycidol	Fish or krill oil	*p*-(dimethylamino)phenol	Aqueous (NaCl solution)	0.5 (ng·mL^−1^, 2/3-MCPD); 0.5 (ng·mL^−1^, glycidoil)	HPLC-MS/MS	This work

## Data Availability

The original contributions presented in the study are included in the article, further inquiries can be directed to the corresponding author.

## Supplementary Materials

The following supporting information can be downloaded at: https://www.mdpi.com/article/10.3390/foods13152340/s1, Scheme S1: Structure of MCPD and glycidol; Figures S1–S4: Derivative characterization; Figures S5 and S6: Demulsification optimization; Figures S7 and S8: Effect of methanol on derivatization reaction; Figures S9 and S10: Optimization of pretreatment conditions; Figures S11–S13: Optimization of derivatization conditions; Table S1: Specification on demulsification methods; Tables S2–S6: Method performance data; Table S7: Glycidol conversion test conditions; Table S8: practical samples test result.
