# Supplementary material for: Determination of Free Glycidol and Total Free Monochloropropanediol in Fish and Krill Oil with Simple Aqueous Derivatization and High-Performance Liquid Chromatography–Tandem Mass Spectrometry
Source: Foods. 2024 Jul 25;13(15):2340. doi: 10.3390/foods13152340 (PMC11311869; doi:10.3390/foods13152340)
Supplement: Supplementary file 1 [file foods-13-02340-s001.zip › foods-3058006-supplementary.pdf]

Article

Determination of Free Glycidol and Total Free Monochloropropanediol in Fish and Krill Oil with Simple Aqueous Derivatization and High-Performance Liquid Chromatography–Tandem Mass Spectrometry

Guangxin Yang [1,†], Yunyu Tang [1,†], Xiaoxia Liu [1,2], Longlong Wang [1,2], Lixia Qin [2], Dan Li [2], Xiaosheng Shen [1], Cong Kong [1,*], Wenlei Zhai [3], Essy Kouadio Fodjo [4] and Chengqi Fan [1]

[1] Key Laboratory of East China Sea Fishery Resources Exploitation, Ministry of Agriculture and Rural Affairs, East China Sea Fisheries Research Institute, Chinese Academy of Fishery Sciences, Shanghai 200090, China; yangpai0717@163.com (G.Y.); tangyunyu@ecsf.ac.cn (Y.T.); yizhangdaodi@163.com (X.L.); longlongwang2022@163.com (L.W.); foodsmc98@126.com (X.S.); fancq@eastfishery.ac.cn (C.F.)

[2] School of Chemical and Environmental Engineering, Shanghai Institute of Technology, 100 Haiquan Road, Shanghai 201418, China; lxqin@sit.edu.cn (L.Q.); dany@sit.edu.cn (D.L.)

[3] Institute of Quality Standard and Testing Technology, Beijing Academy of Agriculture and Forestry Sciences, No. 9 Middle Road of Shuguanghuayuan, Haidian District, Beijing 100097, China; zhaiwl@iqstt.cn

[4] Laboratory of Constitution and Reaction of Matter (Physical Chemistry), Université Felix Houphouet-Boigny, Abidjan 22 BP 582, Côte d'Ivoire; kouadio.essy@univ-fhb.edu.ci

* Correspondence: kongc@ecsf.ac.cn

† These authors contributed equally to this work.

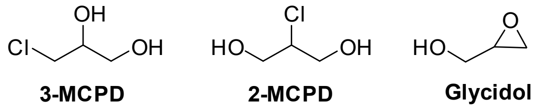

Scheme S1. Structure of 3-MCPD, 2-MCPD and glycidol.

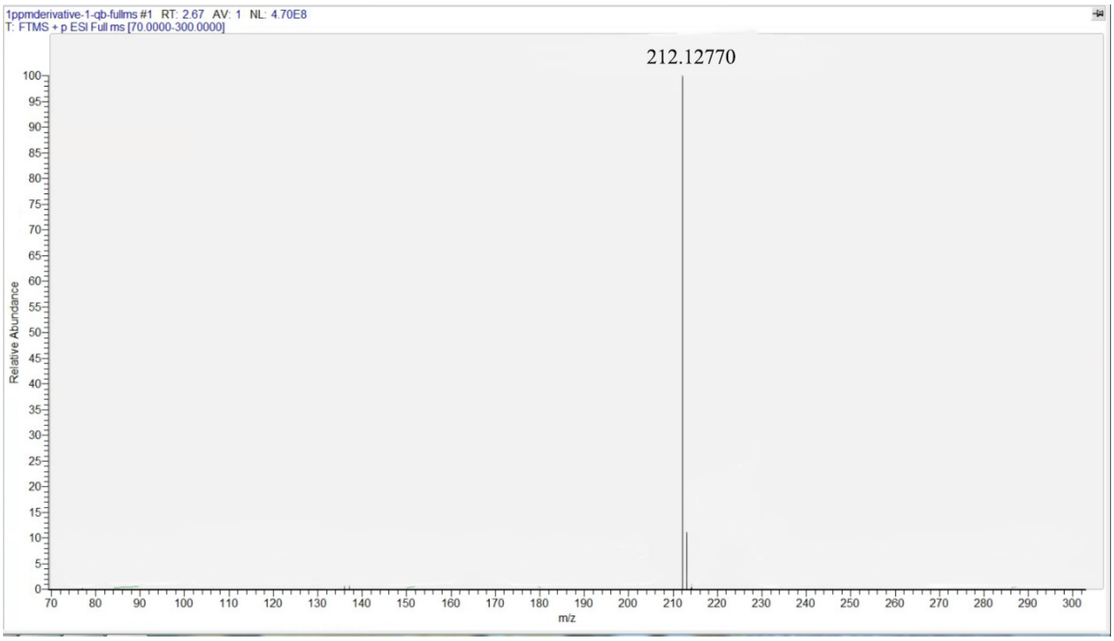

Figure S1. Mass spectrum of **3-PPD**(derivative of 2-MCPD, 3-MCPD and glycidol).

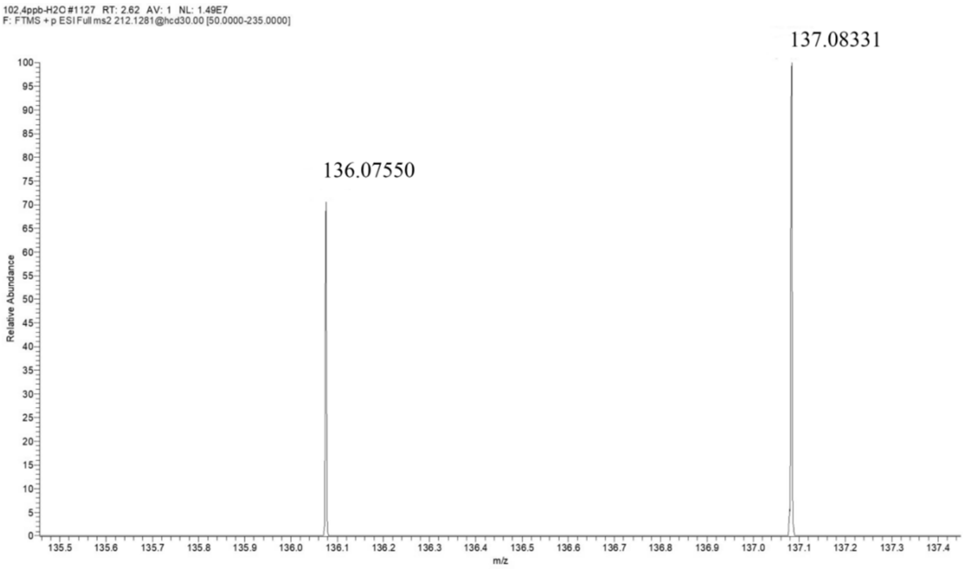

Figure S2. Secondary (fragment ions) mass spectrum of **3-PPD** (precursor: m/z 212.13).

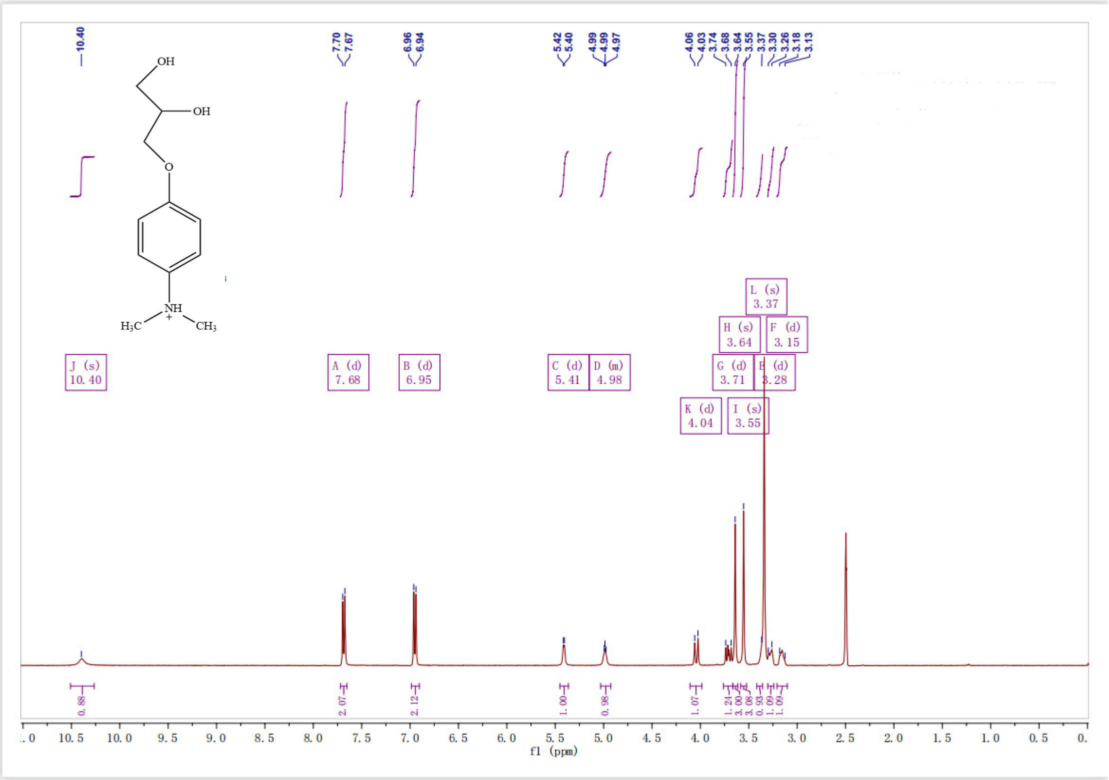

Figure S3. ^{1}HNMR measurement of **3-PPD** (^{1}H NMR (400 MHz, DMSO-d_6) δ10.40 (s, 1H), 7.68 (d, J = 9.3 Hz,2H), 6.95 (d, J = 9.2 Hz,2H), 5.41 (d, J = 4.4 Hz, 1H), 5.03-4.93 (m,1H), 4.04 (d, J = 13.4 Hz,1H), 3.71 (d, J = 22.4 Hz,1H), 3.64 (s, 3H), 3.55 (s, 3H), 3.37 (s, 1H), 3.28 (d, J = 14.7 Hz,1H), 3.15 (d, J = 21.9 Hz, 1H)).

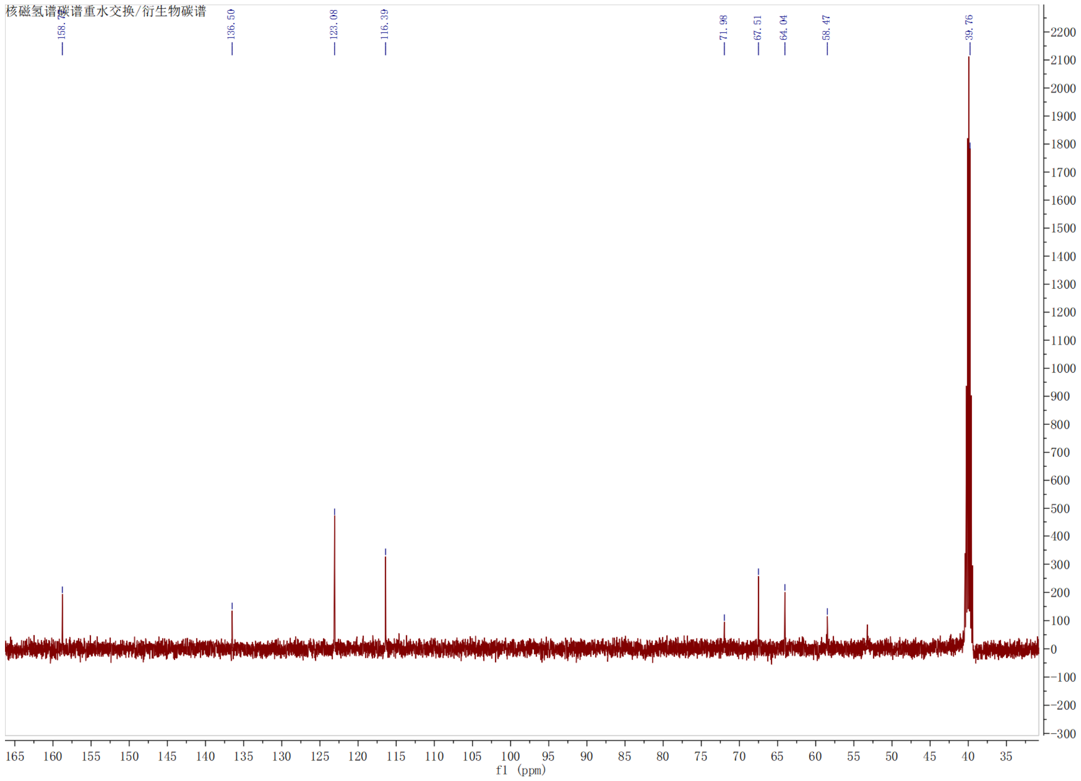

Figure S4. ^{13}C NMR measurement of **3-PPD** (^{13}C NMR (126 MHz, D2O) δ 158.77, 136.50, 123.08, 116.39, 71.98, 67.51, 64.04, 58.47).

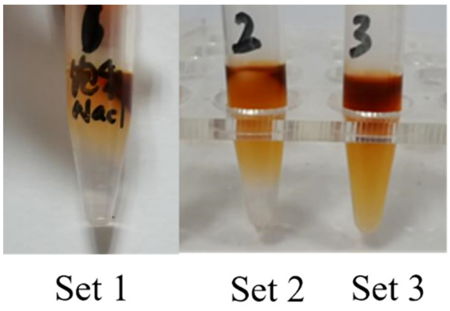

Figure S5. Effect of the demulsification by adding more salt (Set1), temperature variation (Set2), and high-speed centrifugation (Set3) (See Table S1).

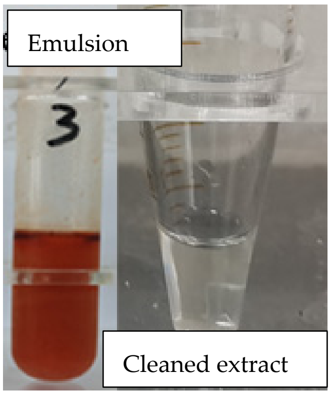

Figure S6. Form of the emulsion during extracting with 10% NaCl solution from the krill oil and the passed solution on the C18 cartridge.

Demulsification

The extract process of MCPD and glycidol in fish oil and krill oil dietary supplements was designed with modification, referring to GB 5009.191-2016, with the aim of simplifying the procedure and reducing operation time. Initially, 20% of NaCl solution was tested for its efficiency in extracting MCPD. It was found that these samples would be obviously emulsified, and would not form distinct water-oil interface after 30 min of silence, especially for krill oil. For this reason, the demulsification process was further examined to obtain a clear extract for subsequent derivatization.

Three different ways of demulsification have been tested, including adding more salt, temperature variation, and high-speed centrifugation (Table S1). Unfortunately, the above-mentioned demulsification methods did not work well to obtain a clear extract for krill oil extract (Figure S5). Therefore, we tested a new way involving passing the emulsified solution through a C18 SPE, as this sorbent can adsorb lipid components and may further break oil-water emulsions. The krill oil (1 mL) was mixed with 20% NaCl solution (3 mL), and vortexed for 10 min. Afterward, 2 mL of the lower sample was passed through the C18 SPE. Finally, a clear extract solvent was obtained (Figure S6).

In order to know the efficiency of emulsification process, 3-MCPD-d_5 (100 ng·mL^{-1}) was added before passing through the C18 SPE. The results showed that 83.4% and 4.5% of 3-MCPD-d_5, respectively, exist in the cleaned extract and effluent of 25% methanol-water (v/v, 2 mL), indicating that no elution was required for this procedure. Moreover, it was found that methanol had an obvious improvement effect on demulsification. Passing the emulsified solution through the C18 SPE was easier after adding methanol (0.5 mL) to the extract (2 mL). However, the lower response values of MCPD-d_5 were also noticed[26], suggesting that the presence of methanol inhibited the derivatization process (Figure S7 and Figure S8).

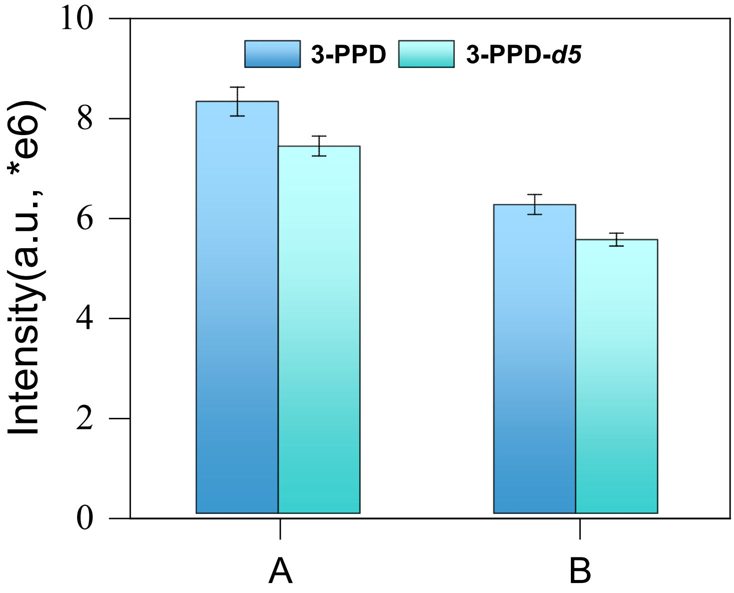

Figure S7. Effect of methanol on 3-MCPD derivatization. (A: 10% NaCl solution; B: 10% NaCl solution (with 20% methanol)).

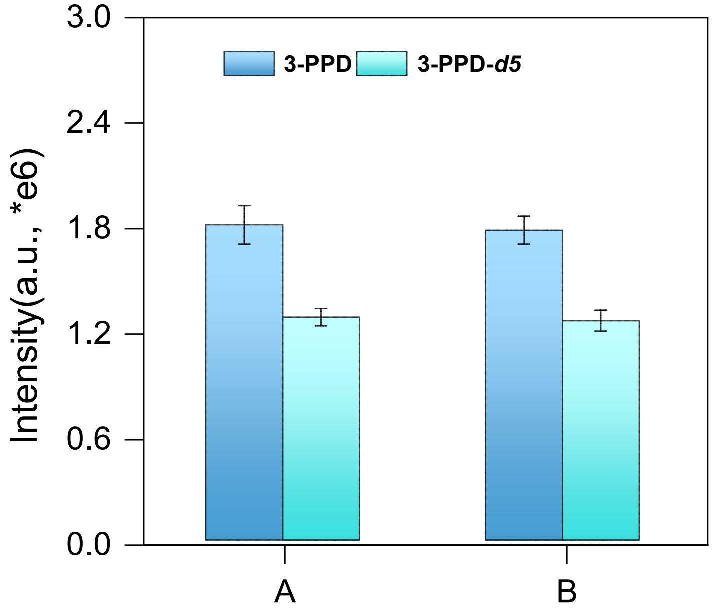

Figure S8. Effect of methanol on glycidol derivatization. (A: 10% NaCl solution; B: 10% NaCl solution (with 20% methanol)).

Furthermore, the column efficiency was examined for 3-MCPD and glycidol. 3-MCPD (100 ng·mL^{-1}) or glycidol (100 ng·mL^{-1}) was added to blank extract solution, followed by passing through the C18 cartridge. On the other hand, the blank extract after passing through C18 was spiked with 3-MCPD (100 ng·mL^{-1}) or glycidol (100 ng·mL^{-1}). The quantification process was done by comparing the response signal of the extract from the spiked sample to the signal of the blank extract added with 100 ng·mL^{-1} of 3-MCPD or glycidol. Afterward, these solutions were derivatized and analyzed in parallel. The column efficiency of the C18 SPE was 98.7% for 3-MCPD and 92.4% for glycidol, indicating an excellent performance in cleanup of the emulsification.

NaCl concentration optimization

The NaCl concentration and the vortex time in the extract process were optimized to improve the extraction efficiency of MCPD and glycidol. Before extraction, krill oil samples were spiked with 100 ng·mL^{-1} of 3-MCPD and glycidol, respectively. Firstly, the sample (1 mL) was extracted with NaCl (3 mL) of different concentrations (0%, 5%, 10%, 15%, 20%, 25%). Then, the extract solution (2 mL) was mixed with methanol (0.5 mL) before passing through the C18 cartridge. In order to avoid the effect of different NaCl concentrations on the derivatization, each obtained extract solution was tuned with water or other blank NaCl solution to get a consistent NaCl concentration (12.5%). Finally, 3-MCPD-d_5 (160 ng) and glycidol-d_5 (160 ng) were added to the solutions, derived following assay A and assay B. The ratio of 3-MCPD/3-MCPD-d_5 was used to evaluate the extraction efficiency. The results showed that the highest extraction efficiency for 3-MCPD was obtained with 10% NaCl solution (Figure S9a), and the extraction efficiency of glycidol was not significantly affected by varying the NaCl concentration (Figure S9b). Therefore, the optimal concentration of NaCl was set at 10%, which can guarantee a high efficiency for both 3-MCPD and glycidol.

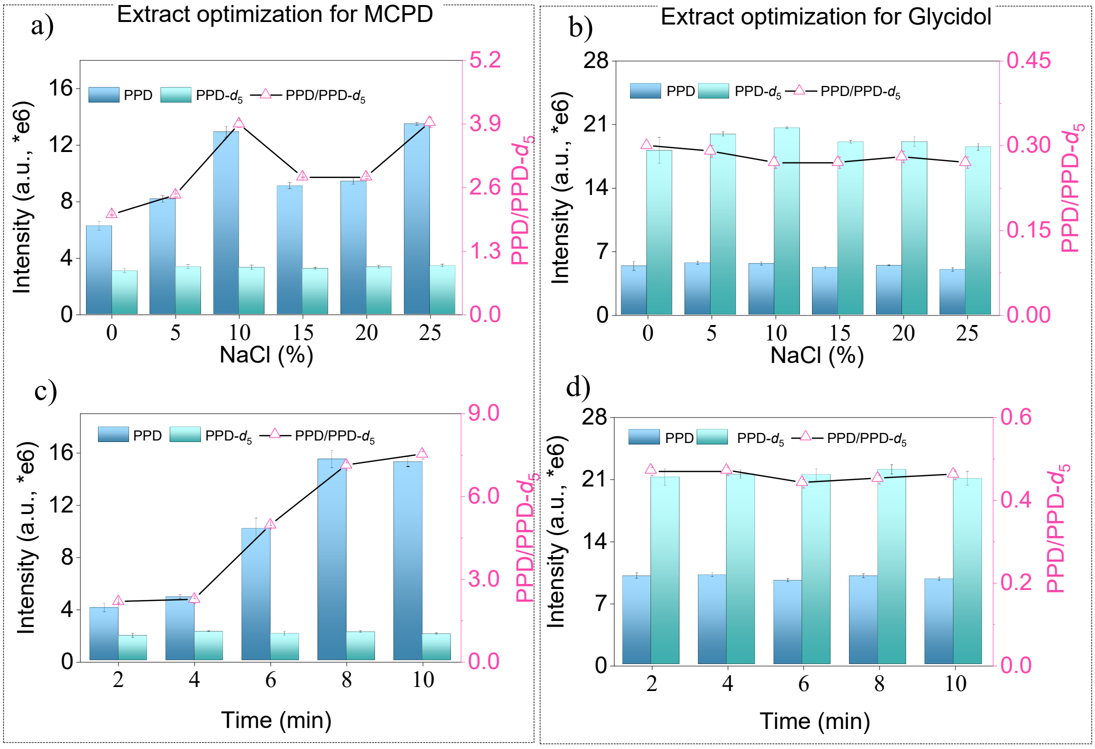

Figure S9. Optimization of pretreatment conditions. a) NaCl concentration for extraction of 3-MCPD; b) NaCl concentration for extraction of glycidol; c) vortex time for extraction of 3-MCPD; d) vortex time for extraction of glycidol.

Furthermore, the vortex time was examined to facilitate the extraction of 3-MCPD and glycidol. The krill oil samples (1 mL) were added with 3-MCPD (100 ng·mL^{-1}) or glycidol (100 ng·mL^{-1}), extracted with NaCl solution (3 mL, 10%) by vortexing from 2~10 min. The extract solution (2 mL) was mixed with methanol (0.5 mL) before passing through the C18 cartridge and was finally derivatized according to assay A and assay B, respectively. Results showed that the krill oil was not completely emulsified after vortexing for less than 4 min (Figure S10a and S10c). However, the extracted 3-MCPD from krill oil reached the maximum level after vortexing for more than 8 min (Figure S9c). The extraction efficiency for 3-MCPD obviously depends on vortex time. Without emulsification, a fairly low extraction efficiency can be observed. Meanwhile, emulsification can enhance at least 2 times of extraction efficiency. Nevertheless, the extraction efficiency for glycidol was not significantly affected by different vortexing time (Figure S9d). Long vortex time resulted in high extent emulsification and led to incomplete cleanup with the used C18 SPE (Figure S10b and S10d), which means a cartridge of more C18 sorbent should be applied. However, the residue oil component in the extract can be removed

through a PTFE membrane. Considering the above results, the optimal vortex time of 8 min was applied to ensure the extraction efficiency of 3-MCPD and glycidol. The above-optimized parameters were also checked on the fish oil, and an extraction efficiency of more than 90% was also observed for the spiking experiment in blank fish oil samples, indicating the suitability of these parameters for both krill oil and fish oil.

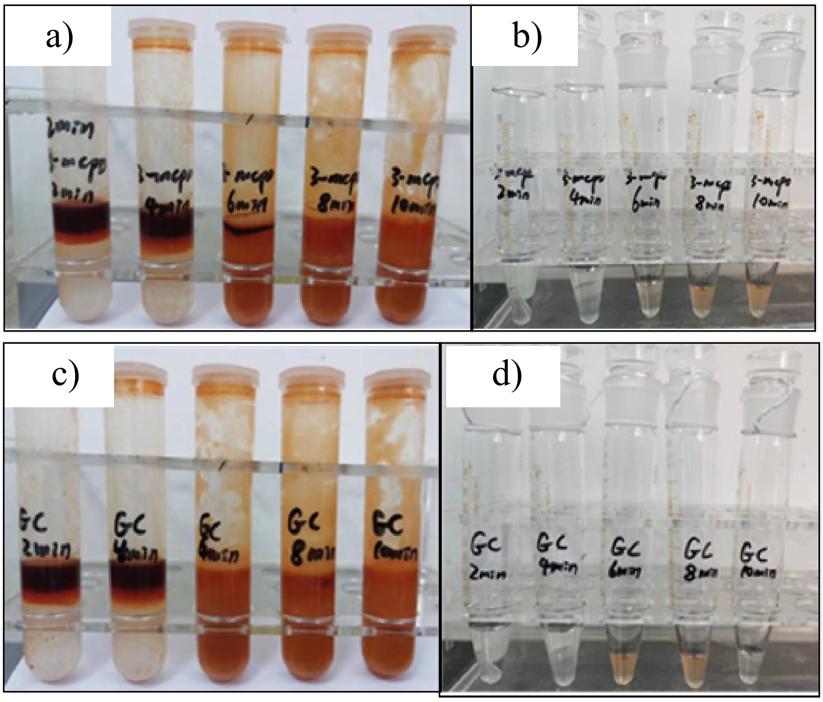

Figure S10. Vortex time optimization (2, 4, 6, 8, 10 min) after mixing krill oil with 10% NaCl 1:3. The emulsion states of krill oil under different vortex times for 3-MCPD (a) and glycidol (c); The extract states after passing the extract (3-MCPD (b) and glycidol (d)) through the C18 column.

Derivatization optimization for the total amount of 3-MCPD, 2-MCPD and glycidol.

Four parameters, including the concentration of *p*-(dimethylamino)phenol and NaOH, derivatization time, and reaction temperature were examined for their influence on the yielding rate of **3-PPD**. The concentration of *p-(*dimethylamino)phenol concentration was varied between 1 ~ 120 $mg \cdot mL^{-1}$. It obviously improved the reaction with the more addition of *p*-(dimethylamino)phenol. The response value of **3-PPD** reached maximum and showed an insignificant increase when the concentration of *p*-(dimethylamino)phenol was higher than 100 $mg \cdot mL^{-1}$ (Figure S11a). The addition of NaOH can facilitate elimination reaction, resulting in the formation of glycidol. It can also affect the further reaction of glycidol with *p*-(dimethylamino)phenol. The addition of NaOH is favorable for the elimination reaction of MCPD, which would further react with *p*-(dimethylamino)phenol. It can be seen from (Figure S11b) that the addition of 1 $mol \cdot L^{-1}$ of NaOH can produce the highest response of **3-PPD**. The more or less addition of NaOH can lead to reduced **3-PPD** production. The response profile of **3-PPD** at different temperatures demonstrated that high temperature could enhance the reaction and further result in high-yield for **3-PPD** (Figure S11c). However, too higher temperature could also lead to evaporation of methanol in the solution, leading to solvent loss during reaction if the container was not well sealed. Therefore, the relatively low temperature (60 °C) would be preferable for the reaction system. The time evolution of **3-PPD** response during reaction was also examined from 0~16 hours. From 0 to 6 hours, **3-PPD** increases with time, and long-time reaction (6~16 hours) shows reduced response of **3-PPD**. Therefore, an increase of more than 6 hours of reaction time would not favor the reaction (Figure S11d). Finally, the optimized parameters for assay A in a practical matrix have been examined at 100

mg·mL^{-1} of *p*-(dimethylamino)phenol, 1 mol·L^{-1} of NaOH, 60 °C and 6 hours, which might achieve a high yield reaction of **3-PPD**.

Assay A was developed for the determination of total amount of 3-MCPD, 2-MCPD, and glycidol. Therefore, 2-MCPD and glycidol should also be evaluated with the above optimized parameters. Equal molar amounts of 3-MCPD (110 ng·mL^{-1}), 2-MCPD (110 ng·mL^{-1}), and glycidol (74 ng·mL^{-1}) were added in three blank krill oil samples, respectively. Meanwhile, 3-MCPD, 2-MCPD, and glycidol were simultaneously added in another blank sample. These four samples were extracted and cleaned with the above-mentioned method and further derivatized under the optimized parameters for assay A. The results showed less than 4% deviation in response signals between 3 samples with single analyte spiking, and the sample with all the 3 analytes addition shows 5% deviation from the sum of the signals with single analyte spiking. It indicated good signal consistency between these analytes, and the derivatization response signals can be used for quantification of each or the sum of them.

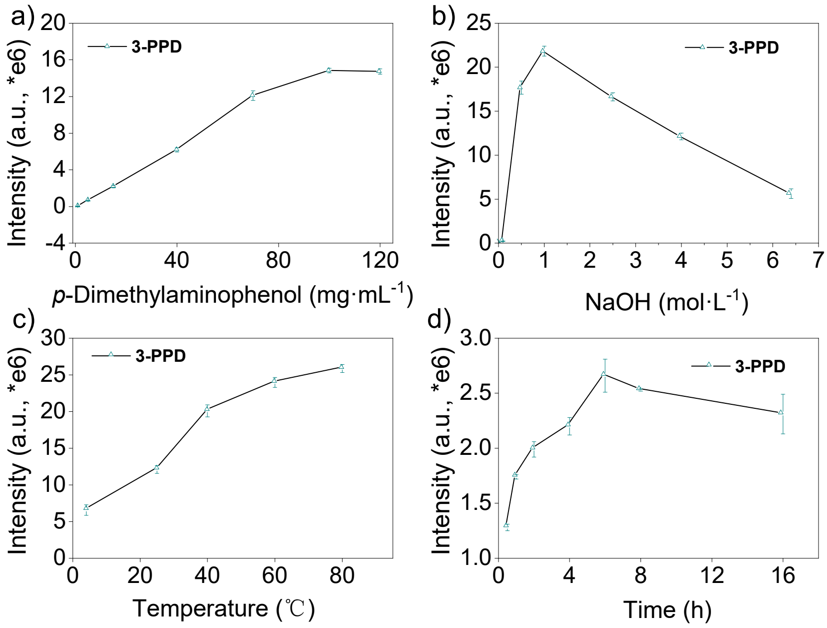

Figure S11. Optimization of derivatization conditions for 3-MCPD. a) *p*-(dimethylamino)phenol concentration; b) NaOH concentration; c) temperature; d) time.

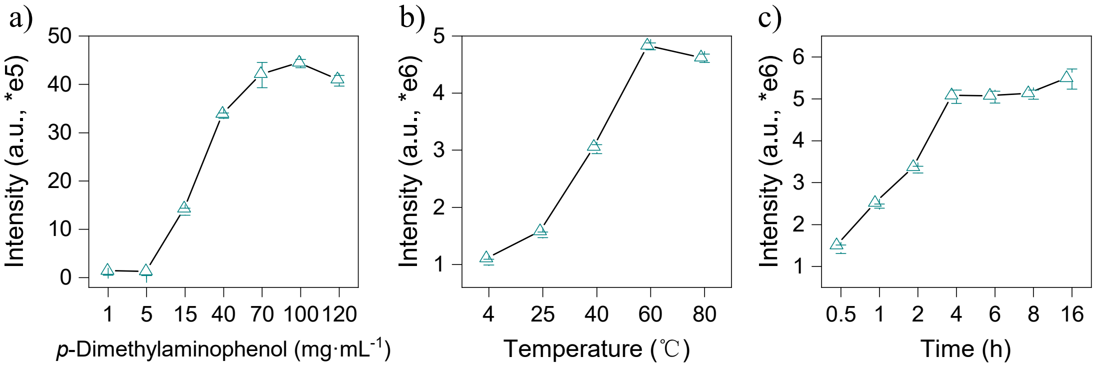

Figure S12. Optimization of derivatization conditions for glycidol (20 ng·mL^{-1}). a) *p*-dimethylaminophenol concentration; b) temperature; c) time.

As shown in Figure S12a, the optimal concentration of *p*-(dimethylamino)phenol for glycidol derivatization is 100 mg·mL^{-1}, showing a stable response between different tests. Similar to the reaction in assay A, the optimum derivatization temperature is 60°C (Figure S12b). Higher heating temperatures can slightly reduce the yielding rate for the derivatization reaction of glycidol, which may be due to the instability of glycidol at higher temperatures. Although the 3-PPD response

value was highest after reaction for 16 h, it is only 10% higher than the response signal at 4 hours (Figure S12c). The reaction may have reached equilibrium at 4 hours. However, to keep the reaction consistent with assay A, a heating time of 6 hours was chosen.

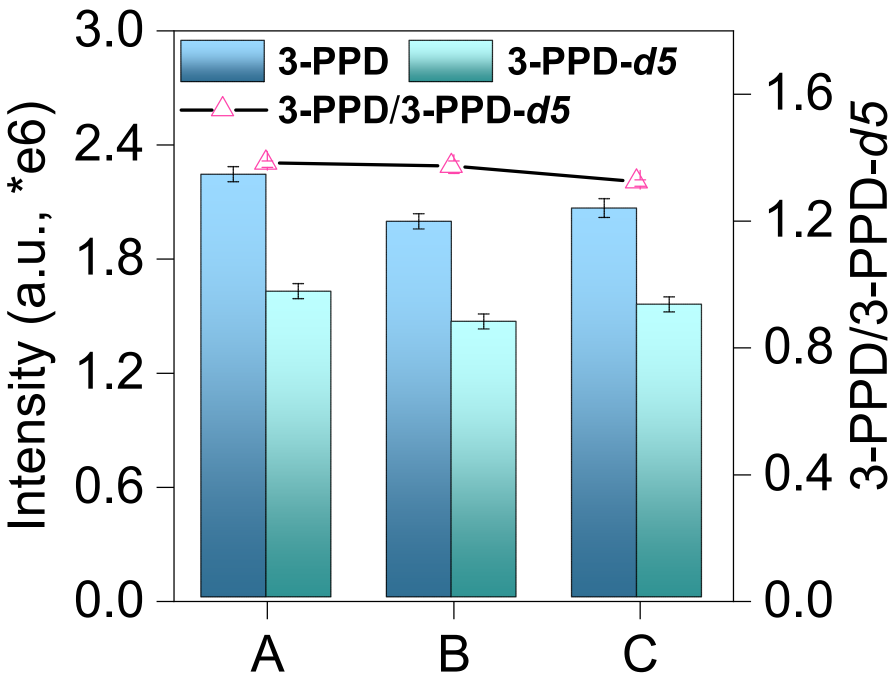

Figure S13. Experimental investigation of whether NaCl extract can induce conversion of glycidol(procedures for A, B, and C are shown in Table S7).

Table S1. Demulsification methods for krill oil in preliminary experiment.

Set	Step 1	Step 2	Step 3	Step 4	Step 5
1	Saturated NaCl	Vortexed for 10 min	-20 °C for 1 hour	Room temperature for 30 min	Centrifuged at 10000 ×g for 15 min
2	20% NaCl solution				
3	20% NaCl solution			Heated at 80 °C for 30 min	

Table S2. Recoveries and precisions of 3-MCPD in fish oil and krill oil (n=3) in spiking experiment.

Substance	Spiked level ($ng\cdot mL^{-1}$)	Recoveries (%)	Average Recoveries (%)	RSD (%)
Fish oil	5	92~99.2	96.3	3.93
	20	109.2~117.2	113.3	3.54
	40	116~119.3	117.4	1.45
Krill oil	200	95.8~102.1	98.3	3.38

Table S3. Inter-batch data for the recoveries of 3-MCPD in fish oil and krill oil (n=3)

Blank samples	Spiked level (ng·mL^{-1})	Day 1 Recoveries (%)	Day 2 Recoveries (%)	Day 3 Recoveries (%)	Average Recoveries (%)	RSD (%)
Fish oil	5	96.3	92.2	117.9	102	13.5
	20	113.3	101.1	111.3	109	6.03
	40	117.4	101.3	113.8	111	7.64
Krill oil	200	97.72	100.7	97.6	98.7	1.77

Table S4. Synchronization experiments for the spiked recoveries of MCPD in fish oil and krill oil (n=3)

Substance	Standards	Spiked level (ng·mL^{-1})	Recoveries (%)	Average Recoveries (%)	RSD (%)
Fish oil	2-MCPD	20	81.8~85.46	83.97	2.29
	3-MCPD		84.82~89.08	87.65	2.79
Krill oil	2-MCPD	200	96.33~102.34	98.38	3.47
	3-MCPD		96.17~100.36	97.72	2.34

Table S5. Spiked recoveries and precisions of glycidol in fish oil and krill oil (n=3)

Blank samples	Spiked level (ng·mL^{-1})	Recoveries (%)	Average Recoveries (%)	RSD (%)
Fish oil	2	89.6~93.2	91.6	2.0
	5	88.96~94.4	92.16	3.1
	20	90.28~96.72	92.95	3.6
Krill oil	2	102.4~114	107.5	5.5
	5	111.7~126.4	118.6	6.2
	20	99.12~105.1	102.3	2.9

Table S6. Inter-batch data for the recoveries of glycidol in fish oil and krill oil (n=3)

Blank samples	Spiked level (ng·mL^{-1})	Recoveries (%)			Average recoveries (%)	RSD (%)
		Day 1	Day 2	Day 3		
Fish oil	2	114.13	113.07	110.53	112.58	1.64

	5	107.89	101.12	106.99	105.33	3.49
	20	97.89	98.69	100.32	98.97	1.25
Krill oil	2	90.13	108.67	100.53	99.78	9.31
	5	102.67	119.52	117.39	113.19	8.10
	20	103.51	112.5	111.27	109.09	4.47

Table S7. Comparison experiments for effect of NaCl solution on glycidol conversion

Set	Procedures
A	Glycidol (50 ng·mL^{-1}) and glycidol-d_5 (50 ng·mL^{-1}) in 10% NaCl:PBS buffer (v/v, 1/1, pH = 6.5) were derivatized with *p*-dimethylaminophenol (100 µL, 100 mg·mL^{-1}), which was kept for 30 min at room temperature, and further incubated for 4 hours in an oven at 60 °C before instrumental analysis.
B	Glycidol (100 ng·mL^{-1}) and glycidol-d_5 (100 ng·mL^{-1}) in 10% NaCl solution was prepared and kept for 2 hours at room temperature, and was then mixed with PBS buffer (v/v, 1/1, pH = 6.5), which was derivatized with *p*-dimethylaminophenol (100 µL, 100 mg·mL^{-1}), and kept for 30 min at room temperature, and further incubated for 4 hours in an oven at 60 °C before instrumental analysis.
C	Glycidol (100 ng·mL^{-1}) in 10% NaCl solution was prepared and kept for 2 hours at room temperature, and was added with glycidol-d_5 (100 ng·mL^{-1}, in PBS buffer (v/v, 1/1, pH = 6.5), which was derivatized with *p*-dimethylaminophenol (100 µL, 100 mg·mL^{-1}), and kept for 30 min at room temperature, and further incubated for 4 hours in an oven at 60 °C before instrumental analysis.

Table S8. Content of glycidol and total MCPD in fish and krill oil samples

Samples	Total MCPD (ng·mL^{-1})	glycidol (ng·mL^{-1})
Fish sample 1	N.D.	N.D.
Fish sample 2	N.D.	N.D.
Fish sample 3	3.44±0.25	N.D.
Fish sample 4	25.26±2.18	N.D.
Fish sample 5	0.99±0.13	N.D.
Fish sample 6	N.D.	N.D.
Fish sample 7	0.95±0.11	N.D.
Fish sample 8	0.668±0.16	N.D.
Fish sample 9	32.78±2.08	N.D.
Fish sample 10	5.26±0.57	N.D.
Krill sample 1	222.32±15.12	5.38±0.47
Krill sample 2	2767.3±72.56	22.20±1.46
Krill sample 3	303.67±18.37	20.01±0.89
Krill sample 4	253.58±14.18	2.46±0.13
Krill sample 5	N.D.	N.D.
Krill sample 6	225.78±10.46	13.53±1.17

Krill sample 7	2174.64±103.45	21.19±1.35
Krill sample 8	135.47±9.87	5.20±0.34
